# Impact of Temperament on Child Behavior in the Dental Setting

**DOI:** 10.5681/joddd.2011.027

**Published:** 2011-12-19

**Authors:** Naser Asl Aminabadi, Firoz Puralibaba, Leila Erfanparast, Ebrahim Najafpour, Zahra Jamali, Sina Ebrahim Adhami

**Affiliations:** ^1^Associate Professor, Department of Pediatric Dentistry, Faculty of Dentistry, Tabriz University of Medical Sciences, Tabriz, Iran; ^2^Assistant Professor, Department of Oral Medicine, Faculty of Dentistry, Tabriz University of Medical Sciences, Tabriz, Iran; ^3^Assistant Professor, Department of Pediatric Dentistry, Faculty of Dentistry, Tabriz University of Medical Sciences, Tabriz, Iran; ^4^Post-graduate Student, Department of Prosthodontics, Faculty of Dentistry, Tabriz University of Medical Sciences, Tabriz, Iran

**Keywords:** Child behavior, dental setting, temperament

## Abstract

**Background and aims:**

Temperament has been suggested to be a predictive factor for the child’s reaction to dental treatment especially in young ages. The aim of this study was to assess the relationship between temperament and child behavior in relation to age in the dental setting.

**Materials and methods:**

In this descriptive-analytical study, 190 children, aged 1 to 7years, who visited dentist for the first time, were included. Early Childhood Behavior Questionnaire (ECBQ) for 18-36 month-olds and Child Behavioral Questionnaire (CBQ) for 3-7 year-old children were completed. Child behavior was rated during dental treatmentusing Frankl scale. Kolmogorov-Smirnov test was used to examine normal distribution of the population. The data were analyzed using descriptive statistics and ANOVA, Chi Square and Pearson’s correlation coefficient. P< 0.05 was considered as sig-nificance level.

**Results:**

58 children (29.6%) had completely negative behavior and 27 children (13.8%) had completely positive behaviorduring the treatment. 65% of the children aged 1 to 3 years had completely negative behavior while this figure was 20% for the children aged 6 to 7 years. Temperament score for the children with completely positive behavior was 257.95 while it was 299.37 for children with completely negative behavior. As the temperament score increased, child behavior rank waslower.

**Conclusion:**

Age and temperament seem to act as predictors of child behavior in the dental setting.

## Introduction

Dentists who treat children often face different forms of avoidance behavior.^1^ Several factors, including psychological and behavioral characteristics, temperament, social status, and age, affect the child’s behavior in different clinical situations, including the dental setting.^2^ Studies have shown that both chronological and intelligence ages have significant influence on the acceptance of dental treatment by children. However, evidence emphasizes the influence of intelligence age.^3 - 5^

Child behavior in the dental setting is a multifactorial phenomenon. Among the factors that influence the child’s behavior is temperament.^3^ Although there is lack of consensus on the definition of the term, most experts refer to it as moods and behavior that originate from the child’s biology and are based on nervous system, manifesting themselves at the early stages of the evolution.^3^

A previous study found a significant difference between the temperament of uncooperative and cooperative children during dental treatment.^6^ Temperamental and difficult behaviors were more frequent in the uncooperative group than in the cooperative group. Children with negative mood and low level of temperament are classified as the high risk, uncooperative child patients.^6^ Therefore, child cooperation in the dental environment seems to be affected in part by the child’s temperament. In this study, we aimed at investigating the relationship between temperament and child behavior during routine dental treatment considering the age factor.

## Materials and Methods

From those referring to the Department of Pediatric Dentistry, Tabriz University of Medical Science, 196 children were selected randomly. Inclusion criteria: First visit to the dentist; age 1-7 years old; presence of carious primary mandibular molars requiring restoration under local anesthesia. A written informed consent was signed by a parent or first caregiver.

Early Childhood Behavior Questionnaire (ECBQ), for evaluating the temperament in 18-36 month-old children and Child Behavioral Questionnaire (CBQ), for the same evaluation in 3-7 year-old children, were completed by parents or caregivers.

ECBQ with 106 items and CBQ with 94 items describe reactions of the children to different situations, and measure their moods and temperament. CBQ items on a scale of “real” and “unreal” with the child reaction during the past 6 months is shown in Table 1. For each question, parent or fist caregiver assigns a score ranging from 1 to 7 or a choice of “non-applicable.” The reliability of these questionnaires with the first and second caregivers has been approved.^7^ The questionnaire was translated into Persian by a psychologist and then back translated into English by a translator. Compared with the original version, necessary corrections were made.

**Table 1:** The scale for determining child behavior being during past 6 months

Scale	Description
1	Extremely untrue of your child
2	Quite untrue of your child
3	Slightly untrue of your child
4	Neither true nor false of your child
5	Slightly true of your child
6	Quite true of your child
7	Extremely true of your child
8	NA (not applicable) when the child has not been seen in those situations

Child behavior during dental treatment was evaluated by a pediatric dentist according to a ranking scale of behavior:

Rating 1; Definitely negative: Refusal of treatment, forceful crying, fearfulness, or any other overt evidence of extreme negativism.

Rating 2; Negative: Reluctance to accept treatment, uncooperativeness, some evidence of negative attitude but not pronounced (sullen, withdrawn).

Rating 3; Positive: Acceptance of treatment; cautious behavior at times; willingness to comply with the dentist, at times with reservation, but patient follows the dentist’s directions cooperatively.

Rating 4; Definitely Positive: Good rapport with the dentist, interest in the dental procedures, laughter and enjoyment.

Data were analyzed using ANOVA, Chi-square, and Pearson’s correlation coefficient. P < 0.05 was considered as statistical significance level.

## Results

Of 196 subjects evaluated, 47.4% were male and 52.6% were female. Age range distribution was as follows: 1-3 year-old, 10.2%; 4-5 year-old, 31.1%; and 6-7 year-old, 58.7%.

Pearson’s correlation matrix revealed a positive significant correlation between age and behavior (correlation coefficient = 0.33, p < 0.001), indicating that a more positive behavior can be expected as the age increases.

In children aged 1-3 years, the frequencies of completely negative, negative and positive behavior were 65%, 30%, and 5%, respectively. None of the subjects showed a completely positive behavior in this age group. The same frequencies for the 4-5 year-old age group were 36.1%, 34.4%, 18%, and 11.5% (completely positive behavior). In 6-7 year-old children, only 20% had a completely negative behavior, followed by 39.1% showing negative, 23.5% positive, and 17.4% completely positive behavior ranks.

Chi-square test showed a significant difference in the frequency of child behavior between age groups (X^ 2 ^= 20.58, p < 0.01).

**Figure 1:**
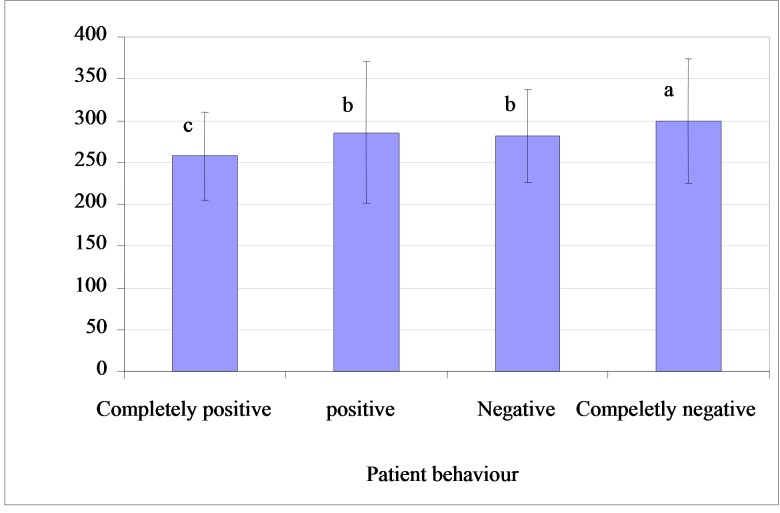
The Frequency of temperament scores based on the behavior type.

The Pearson’s correlation matrix showed a significant negative correlation between child temperament score and behavior (correlation coefficient = 0.168, p < 0.05), indicating that more temperamental and difficult children project more negative behavior. The frequency of temperament scores based on child behavior is shown in Figure 1. In the completely positive behavior, temperament score was 257.9, ranging from 175 to 428. In the positive behavior, temperament score was 285.6, ranging from 140 to 501. In the negative behavior, temperament score was 281.8, ranging from 180 to 492. In the completely negative behavior, temperament score was 299.3, ranging from 185 to 544.

ANOVA variance analysis revealed statistically significant differences in temperament scores of completely positive and completely negative behavior based on the behavior types (p < 0.05), but failed to do so on positive and negative behavior ranks.

Pearson’s correlation matrix showed a significantly negative correlation between the child behavior and subscales of anger, irritability, fear, reaction, reactivity, and shyness.

There was no significant correlation between child behavior and the subscales of activity, emotion, sadness, smile, level of concentration, prohibition, sensation, low and high levels of enjoyment (p > 0.05).

Considering the scores of subscales of anger, irritability, fear, reaction, reactivity and shyness, ANOVA variance analysis showed a significant difference among behavior types, in a way that in completely negative behavior, the average scores of these subscales were significantly higher, while completely positive behavior had lower scores.

In the subscales of emotion, level of activity, sadness, smile and laughter, levels of concentration, prohibition, sensation, low and high levels of enjoyment, there was no significant difference between the score and the type of behavior.

The beta coefficients showed that both age and child temperament, examined by assumption, can be predictors of child behavior. This was not, however, true for the variables of irritability, level of activity, reaction, reactivity, emotion, sadness, smile and laughter, concentration, sensation, low and high levels of enjoyment.

## Discussion

Child cooperation has a critical role in the success of treatment in dentistry. In case of an unpleasant first memory, children will always fear receiving subsequent dental care. Thus, the clinician should be adequately familiar with physical, psychological, educational, and familial status of the child before successful dental treatment.^8^

Personality is an important predictive factor in child behavior. In general, personality is the product of innate abilities and certain behavior related to the immense influences of social and domestic settings. Children with higher degrees of confidence accept dental treatment easier than those who have lower self-confidence.^8^

The types of temperament, as an important factor in child behavior, include active level, anger / frustration, contact / positive, prediction, concentration, collapse, reactivity / ability to calm down, fear, great enjoyment, prohibition, low desirability, sadness, shyness, and smile. ^9, 10^ Child temperament has been used in predicting different aspects of a child’s output. For example, it has been shown that child temperament has a predictor role in the reaction to parent aggression.

The results of the present study show that both of the age and temperament variables can be used as predictors of child behavior in the dental setting. This is in line with the findings of a previous study evaluating the relationship between temperament and sedation by Midazolam and N_2_O and O_2_ gasses, which showed child temperament was effective as a predictor of the success and failure of treatment.^1^

## Conclusion

1. As the child gets older, behavior in the dental setting becomes more positive.
2. There is a negative significant correlation between child behavior and the subscales of anger, irritability, fear, reaction, reactivity, and shyness. Further significant correlations were not, however, seen with subscales of emotion, level of activity, sadness, smile and laughter, concentration, inhibition, sensation, low and high levels of enjoyment.
3. Both variables of age and temperament seem to act as predictors of child behavior in the dental setting.
